# Dietary Patterns Associated with Lower 10-Year Atherosclerotic Cardiovascular Disease Risk among Urban African-American and White Adults Consuming Western Diets

**DOI:** 10.3390/nu10020158

**Published:** 2018-01-31

**Authors:** Marie Fanelli Kuczmarski, Barry A. Bodt, Emily Stave Shupe, Alan B. Zonderman, Michele K. Evans

**Affiliations:** 1 Department of Behavioral Health and Nutrition, University of Delaware, Newark, DE 19716, USA; ejstave@udel.edu; 2 College of Health Sciences, STAR, University of Delaware, Newark, DE 19716, USA; babodt@udel.edu; 3 Laboratory of Epidemiology and Population Sciences, National Institute on Aging, National Institute of Health, Baltimore, MD 21224, USA; zondermana@mail.nih.gov (A.B.Z.); evansm@grc.nia.nih.gov (M.K.E.)

**Keywords:** diet, dietary patterns, diet quality, cardiovascular disease risk

## Abstract

The study’s objective was to determine whether variations in the 2013 American College of Cardiology/American Heart Association 10-year risk for atherosclerotic cardiovascular disease (ASCVD) were associated with differences in food consumption and diet quality. Findings from the baseline wave of Healthy Aging in Neighborhoods of Diversity across the Life Span (HANDLS) study 2004–2009, revealed participants consumed a Western diet. Diet quality measures, specifically the Healthy Eating Index (HEI)-2010, Dietary Approaches to Stop Hypertension (DASH) diet and the Mean Adequacy Ratio (MAR), based on two 24-h recalls collected during follow-up HANDLS studies from 2009–2013, were used. Reported foods were assigned to 27 groups. In this cross-sectional analysis, the participants (*n* = 2140) were categorized into tertiles based on their 10-year ASCVD risk. Lower and upper tertiles were used to determine significantly different consumption rates among the food groups. Ten groups were used in hierarchical case clustering to generate four dietary patterns (DPs) based on group energy contribution. The DP with the highest HEI-2010 score included sandwiches along with vegetables and cheese/yogurt. This DP, along with the pizza/sandwiches DP, had significantly higher DASH and MAR scores and a lower 10-year ASCVD risk, compared to the remaining two DPs–meats/sandwiches and sandwiches/bakery products; thus, Western dietary patterns were associated with different levels of ASCVD 10-year risk.

## 1. Introduction

Cardiovascular disease (CVD) is the leading cause of mortality globally [1]. Dietary patterns, physical activity and genetics affect predisposition to CVD. It has been estimated that the influence of lifestyle factors on CVD mortality amounts to 13.2% for poor diet, 12% for inactivity, and 13.7% for smoking [2]. There is evidence for food-based dietary patterns associated with the potential to reduce CVD risk [2,3,4,5,6]. The Western dietary pattern, described as a diet high in sugar and fat and low in fiber, fruits and vegetables, is associated with a greater risk for developing CVD, compared to a Mediterranean or DASH (Dietary Approach to Prevent Hypertension) eating pattern [7].

Our knowledge about suboptimal intakes of selected foods or nutrients and cardiovascular health is emerging, but is still limited [8,9]. In 2012, Micha and colleagues estimated that 45.4% of annual cardiometabolic deaths were associated with suboptimal food or nutrient intakes. Of the 10 dietary factors they examined, low nuts/seeds and seafood intakes were among the factors associated with the highest proportion of deaths [9]. Some food groups, such as dairy foods, particularly cheese and yogurt, have been reported to lower CVD mortality risk [10,11,12,13,14], suggesting they have protective effects. However, little is known about the effects of intakes of many of these food and food groups when consumed as part of a Western dietary pattern.

Dietary patterns are influenced by demographic and socioeconomic factors. It is recognized that populations with limited income, less than a high school education, and low literacy skills are more likely to consume a Western diet [15]. Prior research with an urban population of African-American and White adults, examined in the Healthy Aging in Neighborhoods of Diversity across the Life Span (HANDLS) study, revealed no dietary pattern consistent with a healthful diet [16]. Amongst this low-income population, measures of diet quality, such as the Healthy Eating Index (HEI)-2010, adherence to the DASH diet, and the Mean Adequacy Ratio (MAR), revealed that major improvements are needed to achieve a healthful diet [17,18].

The objectives of this study were to determine the existence of food group variants in Western dietary patterns associated with lower and upper tertiles of 10-year Atherosclerotic Cardiovascular Disease (ASCVD) risk and to identify dietary patterns associated with lower ASCVD risk.

## 2. Materials and Methods

### 2.1. Healthy Aging in Neighborhoods of Diversity across the Life Span (HANDLS) Study Background

The HANDLS study, a 20-year prospective study initiated in 2004, has been described in detail elsewhere [19]. Participants were drawn from 13 pre-determined Baltimore neighborhoods, yielding a representative factorial cross of four factors: age (30–64 years), sex (men and women), race (African-American (AA) and White (W)), and income (self-reported household income < 125% and ≥125% of the 2004 Health and Human Services poverty guidelines [20], with approximately equal numbers of subjects per factorial cell. Many of the sample with an income ≥ 125% of poverty guidelines were close to the criteria partition [21]. The current cross-sectional study used data from Wave 3, collected in 2009–2013.

There were two interview sessions in the Wave 3 HANDLS study [22]. The first session was completed in mobile research vehicles, located in the participant’s neighborhood or in the participant’s home. This session consisted of a medical history, physical performance assessments, physical examination, cognitive evaluation, laboratory measures, and a first 24-h dietary recall. The second session was done approximately 4–10 days later and consisted of a second 24-h dietary recall and dietary supplement questionnaire completed over the telephone. All HANDLS participants provided written informed consent following their access to a protocol booklet in layman’s terms and a video describing all procedures, and were compensated monetarily. The study was conducted in accordance with the Declaration of Helsinki and the protocol was approved by human Institutional Review Boards at MedStar Health Research Institute and the University of Delaware (Project identification code 129457-13).

**Figure 1:**
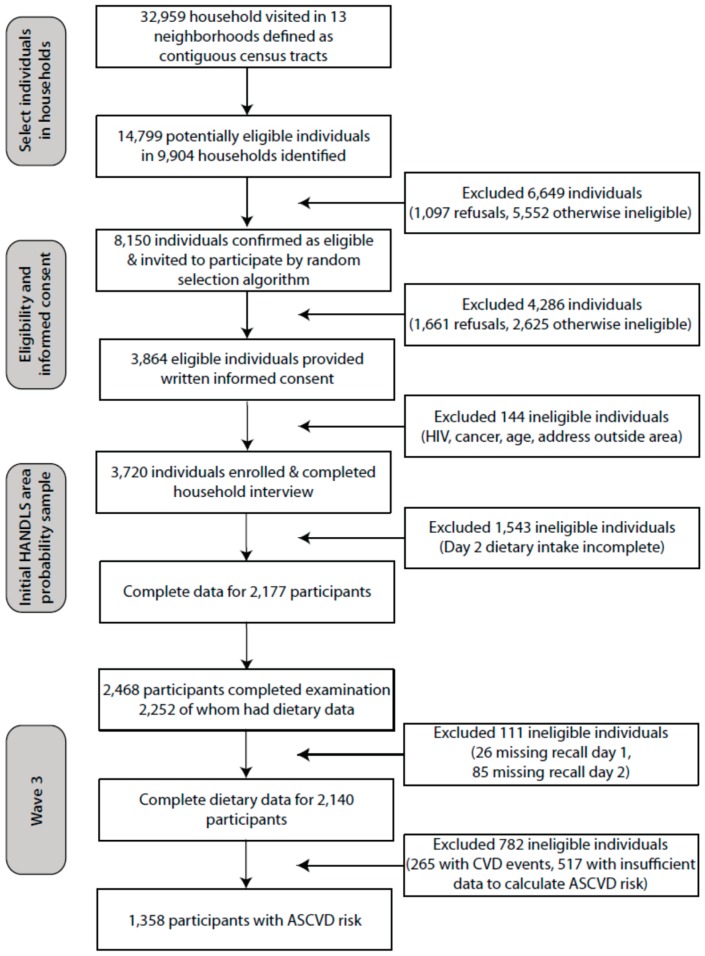
Flow diagram of Healthy Aging in Neighborhoods of Diversity across the Life Span Study household screening, participant eligibility, and response rates. ASCVD: atherosclerotic cardiovascular disease; CVD: cardiovascular disease; HANDLS: Healthy Aging in Neighborhoods of Diversity across the Life Span.

In the baseline HANDLS study, in 2004–2009, a total of 3720 AA and W participants were examined. There were no statistical differences in the distributions of demographic data or energy and nutrient profiles between participants who completed one or both days of dietary recall [16]. Of these participants, 2468 were reexamined in Wave 3. Only 2140 individuals completed two days of 24-h dietary recalls. Of those who had two recalls, 265 participants had CVD events and 1358 participants gave sufficient data to calculate the 10-year ASCVD risk. Those participants with CVD events or insufficient data to calculate the ASCVD risk were excluded from the analyses (Figure 1).

### 2.2. Dietary Method

The United States Department of Agriculture (USDA) computerized Automated Multiple Pass Method (AMPM) was used to collect both 24-h dietary recalls [23]. An illustrated food model booklet, measuring cups, spoons, and ruler were used to assist participants in estimating accurate quantities of foods and beverages consumed. After interviews were completed by trained interviewers, recalls were processed through the post-interview processing system. Then foods were coded using USDA’s Survey Net data processing system to match foods consumed with 8-digit codes in the Food and Nutrient Database for Dietary Studies, version 5.0, and nutrient values were assigned [24].

Foods eaten together were assigned a combination code. Combination codes assigned initially by AMPM were reviewed in Survey Net, providing the coder the ability to change, remove or add new codes to ensure that foods eaten together were correctly linked [25]. Combinations were defined using two separate variables: (i) a combination food number, which distinguishes foods as eaten in combination; and (ii) combination food type. There were 20 combination types—14 were defined by the USDA Food Coding Scheme, specifically beverages, cereal, bread/baked product, salad, sandwich, soup, frozen meal, ice cream, dried beans/vegetable, fruit, tortilla, meat/poultry/fish, lunchables, and chips, and six by HANDLS nutritionists based on the USDA category of “99-other food mixtures”, namely pasta dishes, rice dishes, Chinese dishes, pizza, dairy, and eggs [26]. Examples of combinations include beverages with additions, such as added sugar and dairy products; bread/baked goods with additions, such as jelly to bread; and multi-component items, such as sandwiches and salads. Each food and food combination was assigned to a food group. The nine major food groups in the Food and Nutrient Database for Dietary Studies were expanded to 58 groups to separate groups by their fat, sugar, and sodium contents, as well as by the degree of processing—such as refined versus whole grains. Among the 2140 individuals with two dietary recalls, the percentage who had at least one report of a food was calculated. Using the What We Eat in America food groups as a guide, the 58 groups were reduced to 27 based on similar food type [27] (Supplementary Table S1).

### 2.3. Diet Quality Measures

The score for DASH diet adherence was determined for each participant using the formula reported by Mellen et al. [28]. Mellen et al. identified DASH goals for eight target nutrients, namely total fat, saturated fat, protein, fiber, cholesterol, calcium, magnesium, and potassium. Additionally, sodium was included as a target nutrient even though dietary sodium was held constant in the original DASH study. Micronutrient goals were expressed per 1000 kcal. The total DASH score was generated by the sum of all nutrient targets met. If the participant achieved the DASH target for a nutrient, a value of 1 was assigned, and if the intermediate target for a nutrient was achieved, a value of 0.5 was assigned. A value of zero was assigned if neither target was met. The maximum DASH score was 9; individuals meeting approximately half of the DASH targets (DASH score = 4.5) were considered DASH adherent [28].

Food-based diet quality was also evaluated with the HEI-2010. The National Cancer Institute’s Applied Research website provided the basic steps for calculating the HEI-2010 component and total scores and statistical codes for 24-h dietary recalls [29]. A detailed description of the procedure used for this study is available on the HANDLS website [30]. Component and total HEI-2010 scores were calculated for each recall day and were averaged to obtain the mean for both days combined.

Nutrient-based diet quality was assessed using the Nutrient Adequacy Ratios (NAR) and MAR scores [16]. NAR is defined as the ratio of the participant’s daily intake of a nutrient to his/her current Recommended Dietary Allowance (RDA) for that nutrient [31]. Thus, RDA was adjusted for the age and sex of participants, and vitamin C was adjusted for smokers [32]. Scores for 17 micronutrients—vitamins A, C, D, E, B_6_, B_12_, folate, iron, thiamin, riboflavin, niacin, copper, zinc, calcium, magnesium, phosphorus, and selenium—were calculated. The NAR scores were then converted into a percent with values exceeding 100 truncated to 100. The MAR formula was: MAR = (ΣNAR scores)/17 [16]. MAR was calculated for each recall day and then averaged.

### 2.4. Cardiovascular Risk

Ten-year risk for a first ASCVD is defined as nonfatal myocardial infarction or coronary heart disease death, or fatal or nonfatal stroke, over a 10-year period among people free from ASCVD at the beginning of the period. For Wave 3, the 10-year ASCVD risk was calculated for AA and W men and women aged 40–79 years, using the pooled cohort equations published by the Expert Work Group of the American College of Cardiology and American Heart Association [33]. The variables included in these risk assessment equations included age, total and high density lipoprotein (HDL)-cholesterol, systolic blood pressure, hypertension medication, diabetes, and current smoking status. Among this population, 38% were taking anti-hypertensive medications, 17% lipid lowering drugs, and 10% anti-diabetes medications.

### 2.5. Statistical Analyses

The modeling approach consisted of three stages: (1) reduce the dimensionality of the 27 food groups to a subset of groups that *individually* showed promise in separating cases on the basis of ASCVD risk; (2) use that subset *collectively* to hierarchically cluster cases; and (3) evaluate the resulting dietary patterns (clusters) on their ability to separate cases, not only on the basis of ASCVD risk but also for the other established health indices of DASH, HEI, and MAR. In the first stage, to highlight food group separation potential, tertiles of the 10-year ASCVD risk were calculated and then, using the low (<3.38 ASCVD risk) and high (>8.36 ASCVD risk) tertiles (904 cases combined), food groups were ranked by *t*-test *p*-values, according to significant food group energy differences between the high and low ASCVD risk tertiles. This allowed an objective dimension reduction to 10 food groups for further analysis. Statistical significance (*p* < 0.05) was observed for eight food groups, and results trending toward significance (*p* < 0.10) were observed for two additional food groups. Note that the *p*-value is merely a ranking mechanism in this stage.

**Figure 2:**
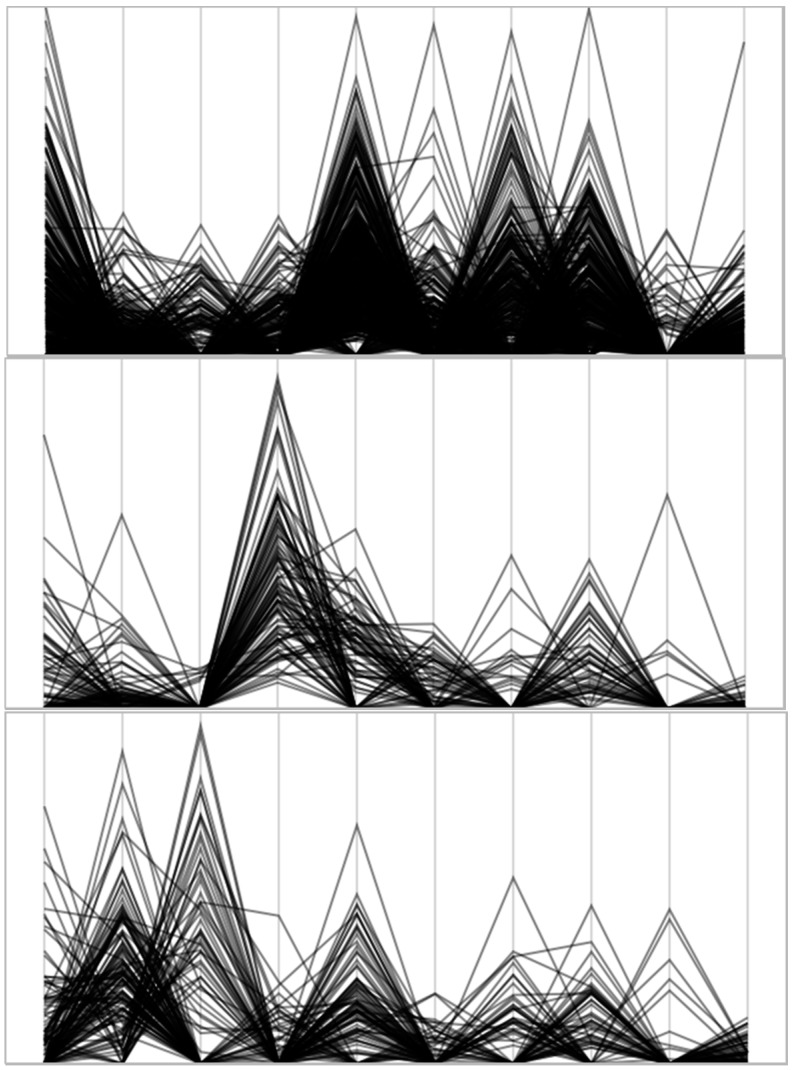

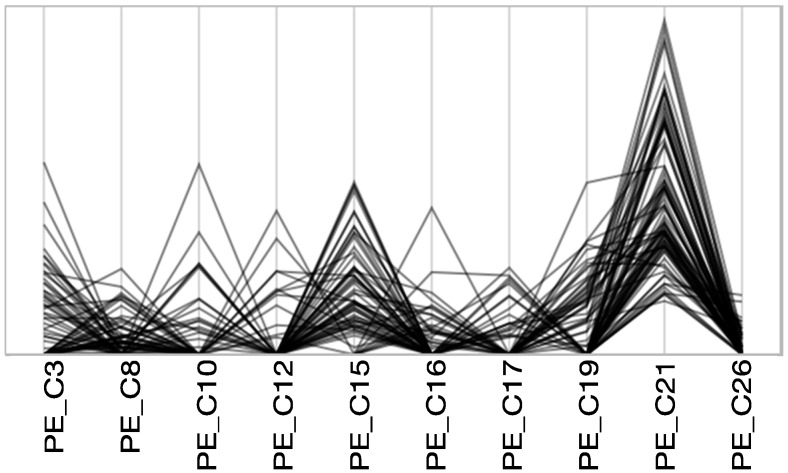
Parallel coordinate plots illustrate the distinct patterns of energy values across the 10 food groups among four dietary patterns. DP1 (top)–DP4 (bottom). Abbreviations: DP1—Dietary pattern 1: Sandwiches/Bakery products, DP2—Dietary pattern 2: Meats/Sandwiches, DP3—Dietary pattern 3: Sandwiches/Other vegetables, DP4-Dietary pattern 4: Pizza/Sandwiches, PE = percent energy; PE_C3—Bakery products; PE_C8—Other Vegetables, excludes dark green, orange and potatoes; PE_C10—Cheese and Yogurt; PE_C12—Meats; PE_C15—Mixed dishes-Sandwiches; PE_C16—Cured meats; PE_C17—Mixed dishes-Meats, poultry, seafood, Mexican, Asian; PE_C19—Eggs and egg dishes; PE_C21—Pizza; PE_C26—Candy and sugars.

In the second stage, the 10 promising food groups became the inputs to a hierarchical clustering of cases routine in SAS JMP^®^ Pro 13.1 (Cary, NC, USA). The algorithm forms natural clusters based on distances between cases computed from the 10-element vector of energy percentages over the promising food groups. Using the Ward distance measure, clusters were formed to minimize the distance between elements within a cluster, while maximizing the distance between clusters. Using a dynamic feature of the software and subjective interpretation of the scree plot, four clusters were retained. Parallel coordinate plots (Figure 2) illustrate the distinct patterns of energy values across the 10 food groups among these four clusters—taken as our dietary patterns (DPs).

The third stage was used to assess how well these DPs explained ASCVD risk, along with DASH, HEI, and MAR. These four measures, taken over the 904 cases, which represent the 452 individuals from each tertile—lower and upper tertiles—served as responses for least square means in linear models. The four DPs were then used as a grouping factor in each linear regression, adjusted for sex, race and income, with Tukey HSD multiple comparisons used for post hoc comparison of means.

## 3. Results

### 3.1. Characteristics of Sample

The mean age of the sample was 53.2 years (*n* = 2140). There were no racial differences in the study sample with respect to age, food insecurity, Body Mass Index, percent of energy from sugar, and intakes of mean energy and protein (Table 1). For both men and women, the percentage of persons with incomes less than 125% of the poverty guidelines and literacy below an eighth grade level, was significantly higher for AA compared to W, and the number of years of education was less. Among men, more W participants smoked cigarettes compared to AA; however, the reverse was observed for women (Table 1).

Diet quality differed by race for the MAR and DASH, with W women having higher scores than AA women for both measures, and W men having higher MAR scores than AA men. There were no significant differences in HEI-2010 scores (Table 1). More W men than AA men used nutritional supplements. Although there was no difference in the percent of men with CVD, the 10-year ASCVD risk was significant higher for AA men compared to W men. More AA women, compared to W women, had CVD and a higher 10-year ASCVD risk (Table 1). Characteristics of the analytical sample (*n* = 1358) are presented in Supplementary Table S2.

### 3.2. Food Groupings and Dietary Patterns

Four DPs were identified based on 10 of the 27 food groups, contributing approximately 39–54% of total daily energy. The top two food groups were used to name the four DPs—sandwiches/bakery products (DP1), meats/sandwiches (DP2), sandwiches/other vegetables (DP3), and pizza/sandwiches (DP4). The percent energy contribution of all 10 food groups, in descending order, is presented in Table 2.

Across all patterns, the first four groups provided at least 30% or more of total daily energy. Sandwiches were either the primary or secondary contributor to total daily energy. They were the major energy contributor for DP1 and DP3. Participants consuming DP3 acquired their next 10% of energy from vegetables other than dark green leafy, orange and starchy vegetables (includes potatoes). Cheese and yogurt provided about 5% energy, ranking fourth among the food groups for persons in DP3. Participants in DP1 acquired their next 7% energy from sweet bakery products, followed by mixed meat dishes and eggs, each contributing approximately 5%. Other vegetables and cheese/yogurt contributed only 1.07% and 0.54% energy to DP1, respectively. Meats (13% energy) and pizza (25%, energy) were the major contributors of energy for DP2 and DP4, respectively. Cheese/yogurt contributed between 0.21–1.12% of energy for these DPs, and other vegetables between 1.44–1.70% energy. Figure 2 uses parallel coordinate plots to illustrate unique DPs. Each case within a DP is a vector-valued measure, graphed in a piecewise linear manner, with endpoints and vertices marking the percent energy value for the corresponding food code. The rise and fall of these lines across the 10 measures represents each case in terms of its energy profile. Similar energy profiles are expected within each dietary pattern, but will differ between patterns. For example, DP4 showed dominant peaks, corresponding to the percent energy from sandwiches (C15) and pizza (C21), whereas the dominant peaks for DP3 were from other vegetables (C8), cheese/yogurt (C10), and sandwiches (C15). In DP1, mixed dishes (C17) is a peak, but is primarily a valley for DP2.

Of the 17 food groups which did not differ by 10-year ASCVD risk, the major energy contributors were breads/grains and cereals (~15%) and sweetened beverages (~11%) (Supplementary Table S3). Alcoholic beverages contributed about 3% of energy for both the lower and upper tertiles.

### 3.3. Diet Quality and 10-Year ASCVD Risk Associated with Dietary Patterns

The mean HEI-2010 scores ranged from 40.80 ± 1.31 for the DP characterized by pizza/sandwiches to 54.82 ± 1.09 for the DP characterized by sandwiches/other vegetables out of 100, the maximum HEI-2010 score. DASH scores, with a maximum of 9, ranged from 1.52 ± 0.14 to 2.31 ± 0.12 for the pizza/sandwiches and sandwiches/other vegetables DPs, respectively. The HEI-2010 and DASH scores for the sandwiches/other vegetables pattern were significantly higher than the sandwiches/bakery products and pizza/sandwiches DPs (Table 3). Although the HEI-2010 score for the sandwiches/other vegetables DP was significantly higher than the meats/sandwiches pattern, there was no difference in DASH scores between those two DPs. MAR scores ranged from 75.89 ± 1.36 to 83.69 ± 1.50 out of 100. MAR scores for sandwiches/other vegetables and pizza/sandwiches DPs did not differ significantly, but were significantly higher than sandwiches/bakery products and meats/sandwiches DPs (Table 3).

Participants consuming the sandwiches/other vegetables and pizza/sandwiches DPs had the lowest 10-year ASCVD risk of the four DPs and were not significantly different. However, they were significantly lower than the remaining DPs. The highest ASCVD risk was observed for the meats/sandwiches DP, which was not different from the risk associated with the sandwiches/bakery products DP (Table 3). There were no significant differences among the DPs with respect to Body Mass Index or waist to hip circumference (data not shown). Energy density, percentage energy contribution of the macronutrients, sex, race, and income by dietary pattern are presented in a Supplementary Table S4.

## 4. Discussion

The study findings contribute to the literature, providing evidence that variations of the Western diet can impact 10-year ASCVD risk. An a posteriori methodological approach was used, but instead of exploring the data to create DPs and then associating the patterns with CVD risk, this study first categorized HANDLS participants by risk and then explored the DPs. On the basis of current evidence, a diet based on the components of the Mediterranean dietary pattern or the DASH pattern lowers risk for CVD [7,34,35,36,37,38,39]. Rank ordering of the DPs by all three diet quality indices revealed that the sandwiches/other vegetables DP had the highest HEI-2010 and DASH scores and the second highest MAR score. As expected, the 10-year ASCVD risk for the sandwiches/other vegetables DP was lower than for the sandwiches/bakery products and meats/sandwiches DPs. This finding is consistent with evidence that better adherence to the DASH plan is associated with lower CVD risk [40]. Sandwiches, defined as a single coded food item and/or consumed as a sandwich combination, were a predominant energy contributor to all clusters. This finding was not surprising, since approximately 49% of the US adult population consume sandwiches daily [41]. While sandwiches were the primary contributor of energy for both the sandwiches/bakery Products DP and the sandwiches/other vegetables DP, the 10-year ASCVD risks were significantly different. The difference could be attributed to the types of sandwiches consumed. Of all the sandwiches consumed by DP1, roughly 23% represent fast food items, with the majority being cheeseburgers (60%). Of the sandwiches reported as combinations of ingredients, 59% contained cured meats. In comparison, 26% of the sandwiches consumed by DP3 were from fast food restaurants, with 40% being cheeseburgers. Approximately 45% of sandwiches identified by combination codes contained cured meats. Cheese was a combination item of many sandwiches in both clusters.

Factors such as energy density of the diet, macronutrient contributors of energy, and/or the demographic characteristics of the people within a DP may also contribute to the ASCVD risk differences. Based on the 10 food groups, the energy density of the sandwiches/other vegetables DP was significantly lower than the sandwiches/bakery products DP (1.25 ± 0.04 vs. 1.47 ± 0.02, *p* < 0.001). (Supplementary Table S3) Compared to the sandwiches/bakery products DP, the sandwiches/other vegetables DP had the highest proportion of women (79%), persons with incomes greater than 125% poverty (71%), and the lowest proportion of AA adults (48%). It is recognized that all three of these demographic factors impact food choices. Women tend to consume more vegetables than men, possibly accounting for the higher percentage of energy from other vegetables [42,43,44]. Socioeconomic disparities in diet quality exist, and groups with lower incomes tend to consume foods that cost less and contain lower nutritional value [45]. In previous research with the HANDLS study, AA participants were found to have lower diet quality than W participants [46]; additionally, pizza was found to be consumed by more W than AA adults [16].

The higher proportion of energy from cheese/yogurt seen in the sandwiches/other vegetables DP might also account for the lower 10-year ASCVD risk compared to the sandwiches/bakery products and meats/sandwiches DPs. Meta-analyses of prospective cohort studies have shown dairy consumption to be associated with a reduced CVD risk [47]. However, there are no clear or consistent patterns for these inverse relationships, suggesting the need for more research. The protective effects of cheese/yogurt, which are rich in calcium, potassium, magnesium, riboflavin, vitamin B^12^, protein, and lauric and myristic fatty acids might explain why they have a beneficial role in CVD reduction [47,48].

Similar to individuals consuming the sandwiches/other vegetables DP, participants in the pizza/sandwiches DP also had lower 10-year ASCVD risk than DPs labeled as sandwiches/bakery Products or meats/sandwiches. Despite having a similarly low ASCVD risk, the energy density between these DPs was significantly different. This finding is consistent with those of Mertens et al. that high-energy dense food products can be part of a DP associated with lower CVD risk [49].

Among the pizzas consumed, 57% were double meat pizzas, 7% meat with vegetables, 26% were just cheese, 7% cheese with vegetables, and 4% chicken or seafood. The MAR score of this DP was higher than the other patterns, but only significantly different from the sandwiches/bakery products and meats/sandwiches DPs. The HEI-2010 and DASH scores of the pizza/sandwiches DPs were lower than the sandwiches/other vegetables DP, suggesting that factors other than diet quality are impacting disease risk. This DP was comprised of more women (63%) and W adults (52%), and more persons with incomes > 125% of the poverty guidelines (68%), compared to the DPs labeled as sandwiches/bakery products and meats/sandwiches.

Meats, specifically beef, pork, and lamb, were the major energy contributor for persons in the meats/sandwiches DP. These individuals had the highest 10-year ASCVD risk. The finding is consistent with reports that meats are associated with higher CVD risk [50]. This DP was comprised of more men (51%) and AA adults (64%), who are known to have higher risks, compared to women and W adults, respectively [51,52,53].

There are many strengths of these analyses. The use of the ASCVD is a strength. The use of this single composite outcome measure, which combines many of the major adverse cardiovascular events, will occur more frequently than its individual components, allowing for detection of associations in smaller sample sizes [54]. Given the complexity of nutrient-disease relationships, this study focused on DPs and their association with CVD risk, representing the current research trend [8]. The patterns are based on two 24-h recalls, which permits better estimation of typical intakes. The incorporation of combination codes allows the researchers to characterize differences in DPs and to distinguish between cheese/yogurt incorporated into dishes and cheese/yogurt eaten separately.

This study also has some limitations. The study was cross-sectional so causal inferences cannot be made. Although two 24-h recalls were used, there is still the possibility of bias due to underreporting [23]. In addition, the hierarchical clustering only used data from the lower and upper tertiles. Cluster analysis allows for opportunities for subjectivity [55]. Investigators make decisions regarding the collapse of the dietary data into smaller number of groups for entry into analysis and the number and type of dietary patterns that are derived. Lastly, the food groups included both regular and reduced fat and sodium items, and whole and refined grains. This grouping may affect the findings. However, the effect for this population could be minimal, since typically less than 5% of the population consumed the more healthful items (Supplementary Table S1).

## 5. Conclusions

In conclusion, this study documented that variants of the Western dietary pattern were associated with different 10-year ASCVD risk. The dietary patterns that are more consistent with the HEI and DASH diet quality indices, even though they were suboptimal quality, were associated with lower ASCVD 10-year risk. At this time the long-term impact of these dietary patterns is unclear; however, future analysis of the longitudinal HANDLS study data might provide answers.

## Figures and Tables

**Table 1:** Characteristics of Healthy Aging in Neighborhoods of Diversity across the Life Span Study Participants, 2009–2013 (*n* = 2140).

Characteristics	Men			Women			*p* Comparison by Race
	African-American *n* = 539	White *n* = 342	*p*	African-American *n* = 776	White *n* = 483	*p*	
**Demographic**							
Age, years, X ± SEM	52.8 ± 0.4	53.8 ± 0.5	0.101	53.2 ± 0.3	53.2 ± 0.4	0.999	0.310
Income, % < 125% poverty	41.7%	26.9%	**<0.001**	48.1%	33.3%	**<0.001**	**<0.001**
Literacy, % < 8th grade	46.5%	27.1%	**<0.001**	45.4%	23.2%	**<0.001**	**<0.001**
Education, years, X ± SEM	12.3 ± 0.1	12.8 ± 0.2	**0.032**	12.4 ± 0.1	12.8 ± 0.2	**0.005**	**<0.001**
**Lifestyle**							
Current Smokers, %	38.3%	59.9%	**<0.001**	43.6%	37.4%	**0.046**	**<0.001**
**Health**							
BMI, kg/m^2^, X ± SEM	28.0 ± 0.3	29.7 ± 0.4	**<0.001**	32.6 ± 0.3	31.5 ± 0.4	**0.024**	0.976
CVD, % with disease	11.6%	11.5%	0.957	15.4%	9.3%	**0.002**	**0.013**
10-year ASCVD risk, X ± SEM	10.65	8.68 ± 0.47	**0.001**	7.02 ± 0.35	4.38 ± 0.24	**<0.001**	**<0.001**
**Dietary**							
Energy, kcal, X ± SEM	2319 ± 41	2418 ± 50	0.124	1792 ± 26	1791 ± 29	0.963	0.257
Protein, gm/kg, X ± SEM	1.07 ± 0.02	1.02 ± 0.03	0.163	0.84 ± 0.02	0.84 ± 0.02	0.951	0.342
Protein, % energy	15.8 ± 0.2	15.2 ± 0.2	0.051	15.6 ± 0.2	14.9 ± 0.2	**0.007**	**0.001**
Carbohydrate, % energy	47.0 ± 0.4	48.1 ± 0.6	0.128	48.9 ± 0.3	50.8 ± 0.5	**0.001**	**0.001**
Sugar, % energy	23.0 ± 0.4	23.1 ± 0.6	0.897	24.7 ± 0.3	24.7 ± 0.5	0.744	0.753
Total fat, % energy	35.2 ± 0.3	35.0 ± 0.4	0.670	35.1 ± 0.3	33.8 ± 0.3	**0.002**	**0.010**
SFA, % energy	11.0 ± 0.2	11.7 ± 0.2	**0.001**	10.9 ± 0.1	11.4 ± 0.2	**0.002**	**<0.001**
MUFA, % energy	12.8 ± 0.1	12.6 ± 0.2	0.323	12.6 ± 0.1	11.7 ± 0.1	**<0.001**	**<0.001**
PUFA, % energy	8.2 ± 0.1	7.6 ± 0.2	**0.005**	8.5 ± 0.1	7.6 ± 0.1	**<0.001**	**<0.001**
HEI-2010, X ± SEM	45.6 ± 0.5	44.9 ± 0.7	0.334	46.5 ± 0.4	47.6 ± 0.6	0.116	0.511
MAR, X ± SEM	78.4 ± 0.6	81.7 ± 0.6	**<0.001**	74.5 ± 0.5	77.2 ± 0.7	**0.001**	**<0.001**
DASH adherence, %	4.4%	3.0%	0.266	4.6%	9.9%	**<0.001**	**<0.001**
Supplements users, %	35.3%	45.3%	**0.003**	48.7%	53.8%	0.077	**0.001**
Food insecure ^1^, %	27.6%	28.7%	0.754	33.8%	30.9%	0.340	0.573

Abbreviations: SEM = standard error of the mean, BMI = body mass index, CVD = cardiovascular disease, ASCVD = atherosclerotic cardiovascular disease risk, SFA = saturated fatty acids, MUFA = monounsaturated fatty acids, PUFA = polyunsaturated fatty acids, HEI = healthy eating index, MAR = mean adequacy ratio, DASH = dietary approaches to stop hypertension, ACC = American College of Cardiology, AHA = American Heart Association. NOTE: 10-year ASCVD risk is based on 2013 ACC/AHA Guideline [33]. ^1^ Defined by affirmative response to question, ‘Did you eat less because of insufficient money for food in the past month?’ Bolded font was used to emphasize *p*-values significant at <0.05 level.

**Table 2:** Percentage of energy contributed by food group, in descending order, by dietary pattern, for HANDLS study participants.

DP1 (*n* = 601) Sandwiches/Bakery Products		DP2 (*n* = 98) Meats/Sandwiches		DP3 (*n* = 124) Sandwiches/Other Vegetables		DP4 (*n* = 81) Pizza/Sandwiches	
Food Group	Energy %	Food Group	Energy %	Food Group	Energy %	Food Group	Energy %
Mixed dishes-Sandwiches	16.06	Meats ^3^	13.02	Mixed dishes-Sandwiches	10.12	Pizza	24.95
Sweet bakery products	6.91	Mixed dishes-Sandwiches	10.30	*Vegetables excludes dark green, orange, & potatoes*	9.87	Mixed dishes-Sandwiches	11.31
Mixed dishes—other ^1^	5.48	Sweet bakery products	4.22	Sweet bakery products	5.05	Eggs and egg dishes	4.48
Eggs and egg dishes	5.19	Eggs and egg dishes	4.15	Cheese and yogurt	4.99	Sweet bakery products	4.47
Candy and sugars	2.47	Cured meats ^2^	2.17	Eggs and egg dishes	2.90	Mixed dishes—other ^1^	1.77
Cured meats ^2^	2.07	*Vegetables excludes dark green, orange, & potatoes*	1.70	Mixed dishes—other ^1^	2.73	Candy and sugars	1.60
*Vegetables excludes dark green, orange, & potatoes*	1.07	Mixed dishes—other ^1^	1.44	Candy and sugars	1.48	Cured meats ^2^	1.55
Meats ^3^	0.88	Pizza	0.68	Meats ^3^	1.00	*Vegetables excludes dark green, orange, & potatoes*	1.44
Cheese and yogurt	0.54	Candy and sugars	0.62	Cured meats ^2^	0.83	Cheese and yogurt	1.12
Pizza	0.41	Cheese and yogurt	0.21	Pizza	0.80	Meats ^3^	0.98

Abbreviations: HANDLS = Healthy Aging in Neighborhoods of Diversity across the Life Span, DP = dietary pattern. ^1^ Includes meat, poultry, seafood, Asian and Mexican mixed dishes. ^2^ Includes cold cuts, bacon, frankfurters, and sausages. ^3^ Includes beef, pork, and lamb.

**Table 3:** Diet quality scores and 10-year risk for Atherosclerotic Cardiovascular Disease (ASCVD) by dietary pattern, adjusted for sex, race, and income for participants in the lower and upper tertiles of risk.

Dietary Pattern	*n*	HEI-2010 ^1^	DASH ^2^	MAR ^3^	10-Year ASCVD Risk ^4^
DP1 Sandwiches/Bakery Products	601	44.93 ± 0.50 ^b^	1.64 ± 0.05 ^b^	76.81 ± 0.57 ^b^	9.18 ± 0.35 ^a^
DP2 Meats/Sandwiches	98	45.30 ± 1.19 ^b,c^	2.17 ± 0.13 ^a^	75.89 ± 1.36 ^b^	9.59 ± 0.85 ^a^
DP3 Sandwiches/Other Vegetables	124	54.82 ± 1.09 ^a^	2.31 ± 0.12 ^a^	81.54 ± 1.25 ^a^	6.30 ± 0.76 ^b^
DP4 Pizza/Sandwiches	81	40.80 ± 1.31 ^c^	1.52 ± 0.14 ^b^	83.69 ± 1.50 ^a^	5.90 ± 0.94 ^b^

Values are Least Squares Adjusted Means ± Standard Error of Mean. Abbreviations: DP = dietary pattern, HEI-2010 = Healthy Eating Index-2010, DASH = Dietary Approaches to Prevent Hypertension, MAR = Mean Adequacy Ratio. ^1^ Superscripts with different letters within a column are significantly different by Tukey HSD multiple comparisons. HEI-2010 score for DP3 is significantly different from DPs 1, 2 and 4 (*p* < 0.001). DP1 is significantly different from DP4 (*p* = 0.0154). ^2^ DASH score for DP3 is significantly different from DPs 1 and 4 (*p* < 0.0001) and DASH for DP2 is significantly different from DP1 (*p* = 0.0005) and DP4 (*p* = 0.0032). ^3^ MAR score for DP3 is significantly different from DPs 1(*p* = 0.0026) and 2 (*p* = 0.0117) and MAR score for DP4 is significantly different from DP1 (*p* < 0.0001) and DP2 (*p* = 0.0007). ^4^ ASCVD includes sex and race within the measure and was only adjusted for income. Risk for DP3 is significantly different from DP1 (*p* = 0.0031) and DP2 (*p* = 0.0208). Risk for DP4 is significantly different from DP1 (*p* = 0.0058) and DP2 (*p* = 0.0192).

## Supplementary Materials

The following are available online at http://www.mdpi.com/2072-6643/10/2/158/s1, Table S1: Percent of individuals with at least one report of uniquely coded food by food group over 2 recall days (*n* = 2140); Table S2: Characteristics of Healthy Aging in Neighborhoods of Diversity across the Life Span Study Participants with 10-year risk for Atherosclerotic Cardiovascular Disease (ASCVD), 2009–2013 (*n* = 1358); Table S3: Comparison of food group consumption by lower or upper tertile of 10-year Atherosclerotic Cardiovascular Disease (ASCVD) risk; Table S4: Characteristics of HANDLS participants in lower and upper tertiles of 10-year atherosclerotic cardiovascular disease risk by Dietary Pattern (DP) (*n* = 904).
