# Supplementary material for: Dietary Patterns Associated with Lower 10-Year Atherosclerotic Cardiovascular Disease Risk among Urban African-American and White Adults Consuming Western Diets
Source: Nutrients. 2018 Jan 31;10(2):158. doi: 10.3390/nu10020158 (PMC5852734; doi:10.3390/nu10020158)
Supplement: Supplementary file 1 [file nutrients-10-00158-s001.pdf]

Table S1: Percent of individuals with at least one report of uniquely coded food by food group over two 24-hour dietary recall days (n = 2140).

Group Number	Food Group	%
1	Breads, grains, and cereals	
	Refined breads/grains	53.9
	100% whole wheat breads/grains	2.1
	Whole grain cooked cereals/crackers	2.4
	Pasta	32.7
	Rice	17.7
	Cooked cereals	15.0
	Ready-to-Eat cereals	30.9
2	Savory snacks	
	Savory snacks, regular	57.2
	Reduced fat snacks	1.1
3	Sweet bakery products	
	Cakes, donuts, pastries	49.9
	Diet cakes/pastries/cookies	0.5
	Fruit desserts	4.6
4	Fruit, excludes citrus fruit	
	Fruit	46.4
	Berries	2.9
	Dried Fruit	1.6
5	Citrus fruit	27.5
6	Dark green and orange vegetables	
	Dark green vegetables	21.2
	Orange vegetables	10.7
7	Potatoes and other starchy vegetables	56.4
8	Other vegetables	45.9
9	Milk	
	Regular milk	9.5
	Reduced fat and fat-free milk	10.8
10	Cheese and yogurt	
	Natural, regular cheese	2.6
	Natural, reduced fat cheese	0.7
	Processed cheese	2.3
	Processed reduced fat cheese	0
	Reg dairy products	5.6
	Low fat dairy products	7.1
11	Dairy desserts	
	Dairy desserts, regular	19
	Low fat and fat-free dairy desserts	2.7
12	Meats	
	Red meats, Lean only eaten	14.8
	Red meats, Lean and fat eaten	15.1
13	Poultry	
	Chicken/turkey prepared with no added fat	10.4
	Chicken/poultry prepared with added fat	40.6
14	Seafood	
	Fin fish prepared with no fat	3.8
	Fin fish prepared with added fat	10.1

	Shellfish	9.4
15	Mixed dishes - Sandwiches	71.1
16	Cured meats	29.4
17	Mixed dishes- Meats, Poultry, Seafood, Mexican American, Asian	
	Meat dishes	30.7
	Diet frozen meals	0.8
18	Soups	16.3
19	Eggs and egg dishes	44.5
20	Legumes	12.7
21	Pizza	13.6
22	Coffee, tea, water	88.2
23	Sweetened beverages	81.4
24	Diet beverages	30.8
25	Alcoholic beverages	22.2
26	Candy and sugar	
	Sugar	3.2
	Sugar substitute	0.7
	Candy	43.9
27	Nuts and nut butters	17.1

Table S2: Characteristics of Healthy Aging in Neighborhoods of Diversity across the Life Span Study Participants with 10-year risk for Atherosclerotic Cardiovascular Disease (ASCVD), 2009-2013 (n=1358).

**Characteristics**	**Men**			**Women**			
	**African American** *n* **= 336**	**White** *n* **= 235**	**p**	**African American** *n* **= 453**	**White** *n* **= 334**	**p**	**P Comparison by race**
**Demographic**							
Age, yrs, X±SEM	53.3±0.4	54.0±0.5	0.281	54.1±0.4	54.2±0.4	0.849	0.397
Income, %<125% poverty	39.3%	24.7%	**<0.001**	47.0%	32.9%	**<0.001**	**<0.001**
Literacy, %<8th grade	45.9%	25.7%	**<0.001**	44.1%	23.4%	**<0.001**	**<0.001**
Education, yrs, X±SEM	12.3±0.2	12.9±0.2	**0.030**	12.4±0.1	12.9±0.2	**0.035**	**0.002**
**Lifestyle**							
Current Smokers, %	63.8%	39.0%	**<0.001**	47.0%	38.8%	**0.003**	**<0.001**
**Health**							
BMI, kg/m^2, X±SEM	27.6±0.3	29.5±0.5	**0.001**	32.0±0.4	30.8±0.5	**0.037**	0.797
10-yr ASCVD risk, X±SEM	10.6± .4	8.7±0.5	**0.001**	7.02±0.35	4.38±0.24	**<0.001**	**<0.001**
**Dietary**							
Energy, kcal, X±SEM	2368±55	2432±58	0.430	1796±33	1821±37	0.622	0.478
Protein, gm/kg, X±SEM	1.09±0.03	1.05±0.03	0.306	0.86±0.02	0.87±0.02	0.781	0.539
Protein, % energy	15.7±0.2	15.3±0.3	0.298	15.7±0.2	14.9±0.2	**0.010**	**0.008**
Carbohydrate, % energy	47.3±0.5	47.3±0.7	0.996	48.6±0.4	50.5±0.6	**.007**	**0.035**
Sugar, % energy	23.2±0.5	22.3±0.7	0.290	24.3±0.4	24.7±0.6	0.664	0.764
Total fat, % energy	34.9±0.4	35.3±0.5	0.651	35.1±0.3	33.9±0.4	**0.030**	**0.175**

SFA, % energy	10.9±0.2	11.6±0.2	**<0.001**	10.7±0.1	11.5±0.2	**0.001**	**<0.001**
MUFA, % energy	12.8±0.2	12.7±0.2	0.776	12.6±0.1	11.8±0.2	**<0.001**	**0.002**
PUFA, % energy	8.1±0.2	7.5±0.2	**0.024**	8.5±0.1	7.6±0.2	**<0.001**	**<0.001**
HEI-2010, X±SEM	45.5±0.6	45.0±0.8	0.606	47.3±0.5	47.6±0.8	0.811	0.933
MAR, X±SEM	78.7±0.7	82.1±0.7	**0.001**	75.3±0.7	77.6±0.8	**0.030**	**<0.001**
DASH adherence, %	2.4%	3.8%	0.316	4.6%	11.7%	**<0.001**	**<0.001**
Supplements users, %	34.1%	45.3%	**0.008**	50.9%	53.5%	0.477	**0.022**
Food insecure[1], %	30.7%	28.7%	0.648	33.0 %	28.0%	0.178	0.187

Abbreviations: CVD = cardiovascular disease, ASCVD = Atherosclerotic Cardiovascular Disease Risk, SFA = saturated fatty acids, MUFA = monounsaturated fatty acids, PUFA = polyunsaturated fatty acids, HEI = Healthy Eating Index, MAR = Mean Adequacy Ratio, DASH = Dietary Approaches to Stop Hypertension. NOTE: 10-year ASCVD risk based on 2013 ACC/AHA Guideline [33]. [1]Defined by affirmative response to question: Did you eat less because of insufficient money for food in the past month?

Table S3: Comparison of food group consumption by lower or upper tertile of 10-year atherosclerotic cardiovascular disease (ASCVD) risk.

**Food Group**	**Lower ASCVD Risk Tertile** *n* = 452	**Upper ASCVD Risk Tertile** *n* = 452	
	Energy,% of total	*Energy,% of total*	*p*
Eggs	3.83±0.29	5.57±0.37	**0.0002**
Vegetables, excludes dark green ,orange and potatoes	2.92±0.25	1.84±0.18	**0.0004**
Mixed dishes - Sandwiches	12.92±0.58	15.48±0.67	**0.0039**
Cheese and yogurt	1.49±0.18	0.84±0.15	**0.0058**
Cured meats	1.50±0.17	2.23±0.22	**0.0088**
Pizza	3.25±0.41	2.13±0.35	**0.0380**
Candy and sugars	2.36±0.25	1.75±0.17	**0.0454**
Sweet bakery products	5.56±0.40	6.74±0.44	**0.0477**
Mixed dishes –Meats, Poultry, Seafood, Mexican, Asian	4.81±0.38	3.86±0.39	**0.0851**
Meats	1.94±0.22	2.50±0.24	**0.0871**
Sweetened beverages	11.57±0.54	10.45±0.45	0.1103
Coffee, tea, water	0.27±0.08	0.15±0.03	0.1571
Legumes	1.31±0.21	1.00±0.16	0.2474
Diet beverages	0.49±0.06	0.60±0.08	0.2620
Nuts and Nut Butters	1.88±0.26	1.51±0.22	0.2739
Breads, grains, and cereals	15.43±0.65	14.52±0.59	0.2954
Soups	1.44±0.23	1.15±0.19	0.3389
Alcoholic beverages	3.50±0.39	3.03±0.35	0.3705
Potatoes and other starchy vegetables	3.95±0.28	4.28±0.26	0.3941
Poultry	5.24±0.37	5.64±0.44	0.4809
Fruit excludes citrus fruit	3.10±0.26	3.31±0.25	0.5586
Citrus fruit	1.26±0.15	1.37±0.15	0.6229
Milk	1.69±0.23	1.54±0.20	0.6356
Dark green and orange vegetables	0.87±0.11	0.81±0.10	0.6416
Savory Snacks	4.55±0.31	4.40±0.33	0.7392
Seafood	2.07±0.25	2.19±0.28	0.7648
Dairy desserts	2.11±0.27	2.21±0.26	0.7804

Table S4: Characteristics of HANDLS study participants in lower and upper tertiles of 10-year atherosclerotic cardiovascular disease risk by dietary pattern (n = 904)

Dietary Pattern	n	Energy/gm food[1]	Protein % Energy[2]	Carbohydrate % Energy[3]	Fat % Energy[4]	Women, %[5]	AA, %[6]	<125% Poverty,%[7]
DP1 Sandwiches/Bakery Products	601	1.47±0.02^{a}	15.01±0.17^{a}	48.55±0.39^{a}	35.34±0.30^{a,}	60.2^{a,d}	62.1^{a}	40.9^{a}
DP2 Meats/Sandwiches	98	1.29±0.06^{b}	18.63±0.57^{b}	45.36±0.11^{b}	33.04±0.79^{b}	49.05^{c}	64.3^{a}	39.8^{a,b}
DP3 Sandwiches/Other Vegetables	124	1.25±0.04^{b}	15.95±0.37^{a}	48.48±0.88^{a,b}	35.16±0.63^{a,b}	79.03^{b}	47.6^{b}	29.0^{b}
DP4 Pizza/Sandwiches	81	1.60±0.05^{a}	15.12±0.33^{a}	48.71±0.97^{a,b}	35.21±0.75^{a,b}	63.05^{a,c,d}	48.1^{b}	32.1^{a,b}

Abbreviations: HANDLS = Healthy Aging in Neighborhoods of Diversity across the Life Span, DP = Dietary Pattern, AA = African American. [1]Superscripts with different letters within a column are significantly different. For energy density, DP1 is significantly different from DP2 (p = 0.008) and DP3 (p < 0.001). DP4 is significantly different from DPs 2 and 3 (p < 0.001). [2] For protein as percentage of energy, DP2 is significantly difference from DPs 1, 3 and 4 (p < 0.001). [3] For carbohydrates as percentage of energy DP2 is significantly different from DP1 (p = 0.017). [4] For total fat as percentage of energy DP2 is significantly different from DP1 (p = 0.023). [5] For sex, the percentage of women in DP3 is significantly higher than DP1 (p < 0.001), DP2 (p < 0.001) and DP4 (p = 0.012). The proportion of women in DP2 is significantly lower than DP1 (p = 0.036). [6] For race, the percent of African Americans in DP3 is significantly lower than DPs 1 (p = 0.003) and 2 (p = 0.013). The percent of African Americans in DP4 is significantly lower than DPs 1(p = 0.016) and 2 (p = 0.030). [7] Fewer participants in DP3 have income <125% poverty guidelines than DP1 (p = 0.013)
